# Novel Biomarkers of Early Atherosclerotic Changes for Personalised Prevention of Cardiovascular Disease in Cervical Cancer and Human Papillomavirus Infection

**DOI:** 10.3390/ijms20153720

**Published:** 2019-07-30

**Authors:** Ingrid Tonhajzerova, Lucia B. Olexova, Alexander Jurko, Bart Spronck, Tomas Jurko, Nikola Sekaninova, Zuzana Visnovcova, Andrea Mestanikova, Erik Kudela, Michal Mestanik

**Affiliations:** 1 Department of Physiology, Jessenius Faculty of Medicine in Martin, Comenius University in Bratislava, 03601 Martin, Slovakia; 2 Biomedical Center Martin, Jessenius Faculty of Medicine in Martin, Comenius University in Bratislava, 03601 Martin, Slovakia; 3 Pediatric Cardiology Martin, Jessenius Faculty of Medicine in Martin, Comenius University in Bratislava, 03601 Martin, Slovakia; 4 Department of Biomedical Engineering, Yale University, New Haven, CT 06520, USA; 5 Department of Biomedical Engineering, CARIM School for Cardiovascular Diseases, Maastricht University, 6200 MD Maastricht, The Netherlands; 6 Neonatology Clinic, Jessenius Faculty of Medicine in Martin, Comenius University in Bratislava, University Hospital Martin, 03601 Martin, Slovakia; 7 Gynaecology Clinic, Jessenius Faculty of Medicine in Martin, Comenius University in Bratislava, University Hospital Martin, 03601 Martin, Slovakia

**Keywords:** human papillomavirus, cardiovascular disease, arterial stiffness, endothelial function

## Abstract

Cervical cancer is associated with a causative role of human papillomavirus (HPV), which is a highly prevalent infection. Recently, women with a genital HPV infection were found to have increased incidence of cardiovascular diseases (CVD), including severe cardiovascular events such as myocardial infarction and stroke. The pathomechanisms of this relation are not yet fully understood, and may significantly affect the health of a large part of the population. Accelerated atherosclerosis is assumed to play a key role in the pathophysiology of this relationship. To identify high-risk groups of the population, it is necessary to stratify the CVD risk. Current algorithms, as widely used for the estimation of CVD risk, seem to be limited by the individual misclassification of high-risk subjects. However, personalised prediction of cardiovascular events is missing. Regarding HPV-related CVD, identification of novel sensitive biomarkers reflecting early atherosclerotic changes could be of major importance for such personalised cardiovascular risk prediction. Therefore, this review focuses on the pathomechanisms leading to HPV-related cardiovascular diseases with respect to atherosclerosis, and the description of potential novel biomarkers to detect the earliest atherosclerotic changes important for the prevention of CVD in HPV infection and cervical cancer.

## 1. Introduction

Cervical cancer is one of the most common malignancies in women, with an estimated 58,300 new cases and 24,400 deaths in Europe in 2012 [1]. The causal factor of cervical cancer is a persistent infection with human papillomavirus (HPV) [2]. HPV infection is the first known necessary aetiological factor of human carcinogenesis. The concept of a “necessary cause” implies that cervical cancer does not and will not develop in the absence of the persistent presence of HPV-DNA. The development of cervical carcinoma is associated with high-risk strains of HPV (HR-HPV), which are capable of the integration of HPV-DNA into the genome of host epithelial cells with consequent expression of viral genes for the production of oncoproteins E6 and E7, and the possible later progression to an invasive stage of the disease [2,3]. Besides the presence of HPV, the formation of cervical carcinoma is conditioned by several other factors, e.g., anatomic localisation of the infection, local cellular and tissue conditions, epithelial proliferative activity, genetic characteristics, and immune processes [2].

The estimated prevalence of anogenital HPV infection in women worldwide was found to be up to 44%, in European women aged 25–35 years about 30%, and the incident infection rate among initially negative women reaches 60% during 5-year follow-up [4,5,6,7]. Despite the unfavourable epidemiological characteristics with a common occurrence of HR-HPV infections, most of the HPV infections are transient and clear spontaneously, and infected women usually do not show evidence of the same type of infection for more than two years [3,4]. Persisting infection may progress to dysplastic changes of the epithelium and, later, to cervical cancer. The process of carcinogenesis takes approximately 12–15 years [3]. Due to a relatively slow progression of cervical intraepithelial neoplasia to carcinoma, good accessibility of cervix uteri for examination, and widely available diagnostic methods, it is possible to detect early precancerous alterations of the epithelium and effectively prevent the development of malignant disease within secondary prevention. Primary prevention in the form of vaccination against HPV infection is also available. Despite the fact that cervical carcinoma is a well preventable disease, its incidence does not decrease in many countries [8].

During recent decades, the progress in treatment has resulted in improved outcomes with a higher survival rate of patients with cervical carcinoma. Nowadays, emphasis is also put on the associated health problems and the potential side effects of treatment, particularly urogenital complications, bowel dysfunction, and psychosocial consequences, such as mood disorders, anxiety, and acute and posttraumatic stress disorder [9]. Paradoxically, much lower attention is drawn to the significantly increased risk of cardiovascular diseases (CVD), which may arise already before the diagnosis and consequent treatment. The issue of cardiovascular risk related to genital HPV infection, precancerous cervical conditions, and carcinoma represents an up-to-date topic which has been scarcely studied. The recently increased incidence of CVD in women infected with HR-HPV points towards an unclear understanding of the pathomechanisms, which may significantly affect the health of a large part of the population [10,11]. Accelerated atherosclerosis is assumed to play a key role in the pathophysiology of this relationship [12,13]. Therefore, the aim of this study is to summarise the current evidence on HPV-related CVD, to review potential direct and associated pathophysiological mechanisms including atherosclerosis, and to emphasise some of the most promising novel biomarkers which could be used in the personalised prevention of CVD in affected individuals.

## 2. Cardiovascular Disease Associated with HPV Infection

### 2.1. Current Epidemiological Evidence

The substantial interest in HPV-related mechanisms which could play a role in CVD has been initiated mostly recently, after epidemiological evidence supporting the relationship between HPV infection and cardiovascular events by Kuo and Fujise [10]. In this population-based cross-sectional study, self-reported data on CVD and self-collected vaginal swabs were obtained from 2450 women, and the specimens were evaluated for the presence of HPV-DNA. Participants with genital HPV infection showed a 2.5-fold increased incidence of severe cardiovascular complications (myocardial infarction, stroke) compared to HPV-negative women; in case of HR-HPV infection, the incidence of these diseases was increased almost three-fold [10]. This association was independent of coexisting medical conditions and traditional cardiovascular risk factors such as smoking, hypertension, obesity, and dyslipidaemia. However, the interpretation of these findings is limited by the cross-sectional design of the study, the unknown temporal relationship between HPV infection and cardiovascular events, self-collection of the specimen, and self-reporting of the data. The assumption of a causative role of long-term HR-HPV infection in an increased risk of CVD has been recently supported by a prospective cohort study, in which a total number of 63,411 women were followed over five years. The prevalence of HR-HPV was 7.6%, HPV positivity was significantly associated with the five-year incidence of CVD, and the hazard ratio for the incidence of CVD in HR-HPV-positive women was 1.23 (95% confidence interval 1.01–1.50) compared to HR-HPV-negative women, adjusted for age, body mass index, smoking status, alcohol intake, physical activity, education level, family history of CVD, cholesterol and glucose levels, and systolic blood pressure (BP) [11]. 

The increased risk of ischaemic stroke and myocardial infarction was also reported in patients with cervical carcinoma after the application of radiation therapy compared to the general population [14]. However, besides the potential effect of HPV infection, it is necessary to consider also the potential local and systemic late side effects of radiotherapy—vascular damage associated with endothelial degeneration, splitting of the basal membrane, deposition of lipids, adventitial fibrosis, and occlusion of the lumen of arteries [14,15]. On the other hand, in patients with malignancy of head and neck after radiotherapy, the risk of strokes was increased four-fold in the case of HPV-positive tumours compared with the patients with HPV-negative tumours [16]. Moreover, a recent case report documented atypical presentation of cervical carcinoma, which was diagnosed secondarily in a 30-year-old patient who was hospitalised due to ischaemic stroke while no traditional risk factors were present [17].

### 2.2. Pathophysiological Mechanisms

#### 2.2.1. Infection-Induced Atherosclerosis

The pathophysiological mechanisms which could potentially determine the associations between HPV infection and CVD are yet not convincingly defined. Regarding CVD, almost 20% of patients with coronary heart disease and other severe conditions don’t have any of the conventional cardiovascular risk factors [18]. Therefore, novel risk factors of CVD have been intensively studied. The effects of chronic infections, which may play an important role in the development, progression, or destabilisation of atherosclerotic CVD, seem to be of particular importance [19,20,21]. This hypothesis is in accordance with the notion of a causative role of the infectious pathogens in the initiation of the atherosclerotic process during infancy and childhood. The adverse effects of these pathogens on the arteries, specifically on the endothelium and smooth muscle, seem to be mediated predominantly by the increased pro-inflammatory activity and altered lipid metabolism [22]. 

In general, pro-atherosclerotic mechanisms related to infections may be local or systemic and direct or indirect. Among the local mechanisms, several pathogenic effects have been considered: increased expression of adhesion molecules, oxidation of low-density lipoprotein (LDL), expression of scavenger receptors promoting increased uptake of oxidised LDL, influx of foam cells, resistance to apoptosis, altered vasomotor tone, and increased production of macrophage chemoattractant protein, growth factors, and matrix metalloproteinases. The systemic effects include the increased release of cytokines stimulating both innate and adaptive immune responses, activation and proliferation of T-helper cells, elevated plasma levels of interferon-alpha, interferon-gamma, tumour necrosis factor-alpha, interleukins IL-1β, IL-6, IL-17, and IL-18, decreased anti-inflammatory properties of high-density lipoprotein (HDL), reactive oxygen species formation, procoagulant activity, and the role of microbial agents and enhanced expression of heat shock proteins in directing the immune response against the host proteins [21,23].

In developed countries, during the last 50 years, the prevalence of atherosclerotic coronary artery disease in young adults declined rapidly, in line with the decline of the prevalence of infectious diseases. The experimental evidence for infection-induced atherosclerosis and the clinical cases of patients with atherosclerotic lesions without traditional CVD risk factors but with a history of severe infections are intriguing [22]. Despite the fact that there is no direct evidence about the influence of antibiotics and viral vaccines on atherosclerosis in humans [22], experimental studies revealed that both antibiotics and viral vaccines have been shown to reduce atheroma in animals [24,25]. This is relevant to the hypothesis that antibiotics and vaccines may independently be the reasons for the decline in the prevalence of atherosclerosis [22]. However, it is necessary to note that the evidence for the role of certain pathophysiological mechanisms of infection-related acceleration of atherosclerosis varies significantly across the studied bacterial and viral pathogens. Specifically, *Chlamydia pneumoniae*, *Porphyromonas gingivalis*, *Helicobacter pylori*, *Cytomegalovirus*, *Influenza virus*, *Hepatitis C virus*, and *Human immunodeficiency virus* (*HIV*) represent the most often infectious agents that have been linked to various pro-atherosclerotic local and systemic mechanisms leading to atheromatous plaques—the higher expression of adhesion molecules (*Chlamydia pneumoniae*, *Cytomegalovirus, Porphyromonas gingivalis*, *Hepatitis C virus*, *HIV*, *Influenza virus),* promotion of LDL oxidation (*Chlamydia pneumoniae*, *Cytomegalovirus)*, the production of macrophage chemoattractant protein-1 (*Influenza virus*, *HIV*, *Cytomegalovirus*, *Chlamydia pneumoniae*, *Porphyromonas gingivalis*), the higher production of growth factors (*Cytomegalovirus*, *Porphyromonas gingivalis)*, the altered vasomotor tone (*Chlamydia pneumoniae*, *Helicobacter pylori*), and the potentiation of systemic inflammation [20,21,22].

#### 2.2.2. HPV as a Candidate Factor for Atypical Atherosclerosis

Compared to other viral infections, e.g., Epstein–Barr virus (EBV), herpes viruses, or cytomegalovirus, current experimental and clinical data supporting the specific role of HPV in atherosclerotic processes are rather scarce [12,20,21]. HPV has been proposed to be a novel candidate factor for atypical infection-associated atherosclerosis based on recent epidemiological findings [10,19]. Since HPV replicates rather locally without the development of classical viremia, the acceleration of the process of atherosclerosis is assumed to be related particularly to the effect of chronic local vaginal inflammation [10,13,26]. The role of the increased levels of circulating inflammatory mediators could be similar to that found in experimental studies of *Porphyromonas gingivalis*. Periodontitis caused by this pathogen resulted in inflammatory cell recruitment in the spleen and caused increased formation of atherosclerotic plaques associated with the accumulation of lipids, macrophages, and T cells [13,27,28]. 

HPV particles with oncoproteins E6 and E7 could, theoretically, be transferred to atherosclerotic plaques through monocytes and macrophages recruited to HPV-infected tissue [13,28,29,30,31]. A recent study showed the presence of HR-HPV DNA in 55% of atheromatous coronary arteries collected from 20 patients deceased due to myocardial infarction [12]. The expression of E6 and E7 oncoproteins was found in arterial smooth muscle cells, plasma cells, foam cells, and macrophages in atheromatous plaques, what may indicate a direct influence of HPV on the structure of the arterial wall [12,32]. HR-HPV DNA was also detected in biopsy samples from the temporal arteries affected by giant cell arteritis, as well as locally in endothelial cells of vessels and in neurons close to cervical carcinoma [12,33,34]. The viral oncoproteins E6 and E7 degrade the tumour-suppressor protein p53, potentially representing an important mechanism linking HPV infection and atherosclerosis, since the protein p53, besides the regulation of cell-division, plays a key role in the regulation of atherosclerotic processes [10,35]. A diminished availability of p53 accelerates atherosclerosis, stimulates the formation of atherosclerotic plaques, and inhibits apoptosis of the infected cells [10,12,36]. In experimental conditions, exposure of the smooth muscle cells of the aorta to the HR-HPV oncoproteins resulted in their prolonged viability, dedifferentiation, and proliferation [32]. Moreover, HPV oncoproteins stimulate nuclear localisation of active caspase 8, an enzyme contributing to the regulation of apoptosis, the inflammatory processes in the vessels, and atherosclerosis itself [12,37,38]. Besides these mechanisms, a chronic HPV infection may be associated with impaired lipid metabolism, what constitutes another potential factor for the acceleration of the atherosclerotic process [39,40]. 

Several studies referred to the link of a defect in lipid metabolism to CVD including hypertension [41,42]. Previous studies noted that hypertension is characterised by multiple alterations in the structure and function of the cell membrane, including changes in membrane microviscosity, receptor function, signal transduction, ion transport, calcium mobilisation, or intracellular pH regulation. In this context, lipids, as an integral part of the cell membrane, play a crucial role in the modulation of the membrane properties [43]. Thus, hypertension-linked impaired lipid profile characterised by elevated plasma triglycerides, low HDL cholesterol, and increased LDL levels could contribute to membrane abnormalities [42,43]. With respect to HPV infection, Brito et al. [44] found lower HDL and higher systemic blood pressure levels in HPV-positive women compared to HPV-negative women. It seems that HPV-linked impaired lipid profiles could represent a risk factor for hypertension.

### 2.3. Other Risk Factors Associated with CVD and Carcinogenesis

CVD and cancer represent the two major causes of premature mortality worldwide and share several risk factors, biological mechanisms, and their potential interactions [45]. Regarding atherosclerosis and CVD in general, smoking is considered a major risk factor which consistently shows significant strong association with the risk of severe cardiovascular events [46]. The common pathophysiological mechanisms for both carcinogenesis and CVD include the production of pro-inflammatory and oxidising substances, irritants, and carcinogens, and the potential effect of nicotine on the inhibition of apoptosis and stimulation of angiogenesis [45]. Furthermore, endogenous sex hormones (oestrogens, progesterone) in premenopausal women show cardioprotective effects which result in lower incidence of CVD; however, the beneficial effect of hormonal replacement therapy has not been sufficiently proven and remains controversial, while it is also associated with the adverse effects on the risk of thromboembolism [47]. The current use of oral contraceptives was found to be significantly associated with the risk of venous thromboembolism and ischaemic stroke; however, discontinuation of use seemed to result in a rapid return to baseline CVD risk [48]. Sex hormones seem to be a necessary factor for the progression of vaginal HPV infection to malignant disease, and greater exposure to sex hormones, e.g., due to prolonged oral contraceptive use, is associated with increased risk of cervical cancer [49]. Additionally, low socioeconomic status is a well-known risk factor for a broad spectrum of diseases, including cervical cancer and CVD, in particular, those related to atherosclerosis [49,50].

### 2.4. Current Concepts of Atherosclerotic Mechanisms

Although the atherosclerotic process is not fully understood, recent data showed that inflammation couples dyslipidaemia to atherogenesis initiated by inflammatory processes in the endothelial cells of the vessel wall in response to LDL particles. In other words, early atherogenesis is characterised by expression of pro-inflammatory cytokines and leukocyte recruitment. Furthermore, the inflammatory pathways promote thrombosis that may lead to myocardial infarctions and strokes [51,52]. Thus, identifying inflammation triggers a better understanding of the inflammatory pathways may provide a window into potential new therapeutic targets. 

In this context, increased intima-media thickness (IMT) is associated with atherosclerosis development and progression [53]. With respect to inflammation, it is supposed that IMT is strongly related to circulating levels of pro-inflammatory IL-17-related chemokine (eotaxin). Specifically, the association between the visceral fat and circulating eotaxin levels on the one hand, and IMT on the other hand, could reinforce the hypothesis that IL-17, released by the macrophages of visceral adipose tissue, induces eotaxin secretion via the smooth muscle cells present in the atheromatous vessels [54]. Moreover, IL-17 induces endothelial cells apoptosis by activating caspase-3 and caspase-9, potentially leading to vascular endothelial damage [55]. Taken together, the IL-17 as a key cytokine regulating local tissue inflammation could play an important role in pathways/mechanisms triggering early atherosclerotic processes due to the strong association between IL-17 and eotaxin circulating levels [4]. 

Recently, high-risk HPV infection could play an additive or synergic role to promote CVD development, mainly in individuals with obesity and metabolic syndrome (MetS) [56]. Notably, MetS was found to be associated with an increased risk of persistent HPV infection in the presence of obesity [57]. This association could be explained in a manner that abnormal metabolic milieu associated with the ineffective immune response against HPV infection may result in persistent HPV infection and increased viral replication enhancing the HPV-induced systemic inflammatory response. In other words, obesity and MetS may adversely affect immunity, as well as pathogen defence (e.g., lymphoid tissue integrity disruption, alterations in leukocyte development, coordination of adaptive immune responses) [58,59]. Additionally, obesity is associated with increased oxidative stress, endothelial dysfunction, metabolic abnormalities, and increased pro-inflammatory cytokines. From this perspective, altered immune response in obesity and MetS, and increased levels of abnormal adipokines and inflammatory markers in persistent HPV infection may contribute to CVD development [58,60,61,62]. 

Importantly, there is a high demand for identifying novel potential molecular markers as effective anticancer therapy targets. In this aspect, the SRY-related HMG-box 18 (SOX18) protein as an important transcription factor involved in the vascular development during embryogenesis has been proposed to be a significant diagnostic and prognostic marker in various cancer types including cervical cancer. Specifically, the SOX18 protein is involved in the Hedgehog signalling pathway in non-cancerous cells during embryogenic development. Despite the fact that the Hedgehog pathway remains inactive in the mature human cells, its activation has been shown in cervical cancer. Therefore, modulation of this pathway by inhibitors may be important in future-targeted anticancer therapy [63]. Regarding cardiovascular risk, the SOX18 protein could play an important role in atherogenesis involving cell growth processes such as arterial intimal thickening and neovascularisation [64]. Moreover, microRNAs (miRNAs), exactly miRNA221 and miRNA222 playing a crucial role in vascular smooth muscle cells proliferation, could represent other potential novel biomarkers for diagnosis of atherosclerosis [65].

It seems that the current understanding of the direct and indirect pathomechanisms leading to atherosclerosis could be crucial in identifying new potential therapeutic targets to prevent or decrease CVD and cancer risk. 

**Figure 1:**
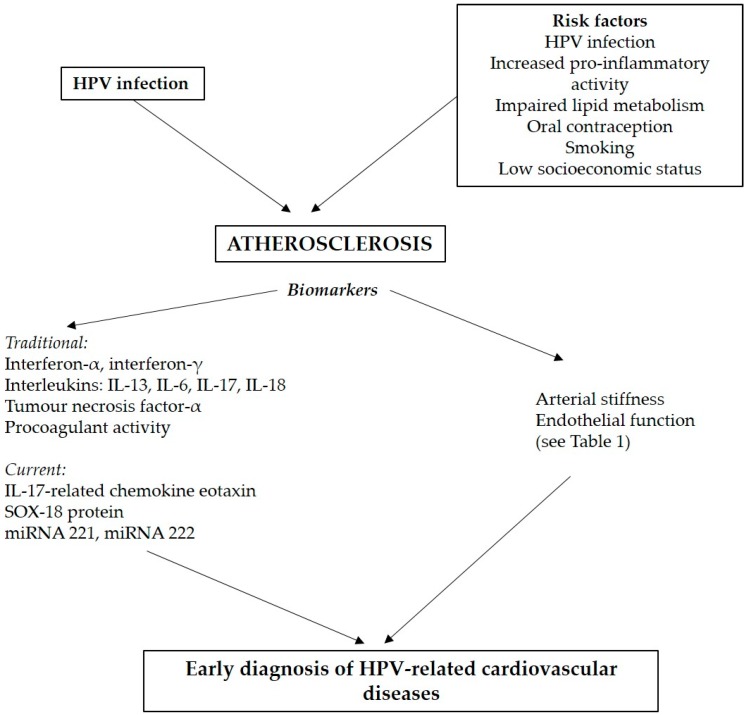
Schematic diagram of atherosclerosis-linked different risk factors and biomarkers HPV—Human Papilloma Virus; SOX-18 protein—SRY-related HMG-box 18, miRNA—microRNA.

A summary of the discussed atherosclerosis-linked risk factors and biomarkers is given in Figure 1.

## 3. Methods for Personalised Prediction of Cardiovascular Risk

The stratification of CVD risk is a key tool for the successful identification of high-risk groups of the population and the consequent application of the effective interventions to minimise cardiovascular morbidity and mortality [46]. Today, several algorithms for the estimation of CVD risk are used, e.g., the Atherosclerotic Cardiovascular Disease risk score from the American Heart Association and the American College of Cardiology [66], United Kingdom QRISK2 model [67], European SCORE risk estimator [68], Framingham risk score [69], and others [70]. The distinct CVD risk estimators differ in the source population, the number of the evaluated parameters, predictive ability of fatal/non-fatal CVD events, and associations with the efficacy of certain therapeutic strategies [46,70]. The most commonly evaluated risk factors are age, smoking status, blood lipid levels, and systolic BP; less frequently used are, e.g., family history of CVD, sex, diabetes mellitus, body mass index, and ethnicity [46].

So far, all of the widely used conventional CVD risk algorithms have been developed for the population-based prediction of CVD (i.e., of the number of subjects within the population who will develop CVD over a certain time period), and their major limitation seems to be a considerable individual misclassification of high-risk subjects [46]. Yet, the generally accepted evidence-based personalised prediction of cardiovascular events is missing. This situation could be improved by the identification of novel biomarkers, which are sensitive to individually assess the total effects of numerous mutual combinations of risk factors [71,72]. Regarding the issue of HR-HPV-related CVD risk with the assumed key role of atherosclerosis, biomarkers reflecting the early atherosclerotic changes could be of major importance for the future efforts in the personalised prediction of cardiovascular events in HPV-infected individuals [12,13].

### 3.1. Evaluation of Early Atherosclerotic Alterations

The term atherosclerosis involves two distinct processes affecting the arteries as a consequence of ageing and the effects of cardiovascular risk factors—atheromatosis and arteriosclerosis. Atheromatosis is characterised by low-grade vascular inflammation, infiltration of macrophages and leukocytes into the arterial wall, excessive local oxidation of low-density lipoproteins, and, consequently, dysfunction of the endothelium. Endothelial dysfunction represents an early, often reversible stage of arterial damage, which precedes the irreversible chronic inflammatory changes associated with calcification and scarring [73]. Arteriosclerosis describes loss of the elasticity of the arterial wall due to an increased volume of extracellular matrix, hypertrophy of vascular smooth muscle cells, degradation of elastin, deposition of stiffer collagen fibres and calcium, and, under certain conditions, extensive formation of advanced glycation end-products, which cross-link the collagen molecules resulting in a functionally stiffer collagen matrix [74,75,76]. Thus, arteriosclerosis could be defined as a diffuse, non-occlusive process characterised by impaired material properties of the load-bearing components of the arterial wall, and altered neuro-humoral regulation and cell signalling pathways determining vascular structure and functions [73,75]. Atheromatosis and arteriosclerosis often coexist, and there are several mutual interactions between the underlying mechanisms of both processes [73,77]. Today, modern methods for the assessment of endothelial function and arterial stiffness offer evaluation of the first detectable markers reflecting the pathological effects of cardiovascular risk factors (e.g., elevated BP, smoking, hyperlipidaemia, physical inactivity, mental stress) and the consequent atherosclerotic damage already at early, clinically silent, stages [78]. 

#### 3.1.1. Endothelial Function

The endothelium represents a smart controller of blood flow with diverse biological roles in the micro- and macrovascular circulation, which is modulated through the release of paracrine, autocrine, and endocrine vasoactive substances in response to physical and chemical stimuli [79]. Endothelial dysfunction is defined as a state of imbalance between the factors with vasodilatory, antithrombogenicity, and antimitogenic effects (mainly nitric oxide (NO), prostacyclin, and endothelium-derived hyperpolarising factor), and substances with vasoconstrictor, prothrombogenic, and proliferative effects (e.g., angiotensin II, endothelin 1, free oxygen radicals, and thromboxane) [80,81]. It is a systemic condition characterised particularly by a decreased bioavailability of NO, is associated with many cardiovascular risk factors and conditions predisposing to atherosclerosis and CVD, and is considered to reflect the total burden of all the involved pathophysiological mechanisms [82,83]. Impaired endothelium-dependent vasodilation is a surrogate marker of arterial damage, which typically precedes the morphological changes, and thus allows for early assessment of atherosclerotic changes, at a stage where these are still reversible [73,80,84].

Currently, the non-invasive evaluation of endothelial function is most often performed using ultrasound evaluation of flow-mediated dilation in the brachial artery (FMD), or using plethysmographic evaluation of the finger pulse wave amplitude using peripheral artery tonometry (PAT). In both methods, firstly, the baseline resting parameters are recorded. Then, blood flow to the evaluated arterial bed is temporarily occluded using a cuff (typically for five minutes). After the release of the cuff and restoration of blood flow, the consequent reactive hyperaemia is evaluated as a post/preocclusion change in artery diameter (FMD) or pulse wave amplitude (PAT). While in FMD, the changes of the diameter of the brachial artery are evaluated from a single side recording, in the PAT method, plethysmographic curves are recorded simultaneously at the index fingers of both hands, and the occluded and non-occluded sides are compared. This method allows monitoring of the contralateral changes in vasoconstriction and the consequent correction of the recorded parameters of the reactive hyperaemia, and diminishes the observer error [80]. 

Importantly, FMD and PAT may offer information about distinct aspects of endothelial function, which cannot be interpreted fully interchangeably [79,80]. FMD evaluates the macrovascular endothelial function, is predominantly (although not exclusively) dependent on the bioavailability of NO, and its predictive power for the estimation of cardiovascular risk is assumed to be greater in patients with already-developed atherosclerosis [79,80,85]. Reactive hyperaemia assessed by PAT seems to be mediated by the release of NO to approximately 60% with rest of the effect potentially attributable to the regulation by the autonomic nervous system, and its application seems to be more significant in younger individuals with a lower degree of atherosclerotic changes [79,80]. However, these characteristics should not be considered as a strict delineation of the application of the individual methods since the sensitivity to assess the effects of specific risk factors in certain populations is still not fully explored. For example, FMD was found to be a sensitive method to assess impaired endothelial function in otherwise healthy adolescents with newly diagnosed white-coat and essential hypertension [86].

#### 3.1.2. Arterial Stiffness

Arterial stiffness reflects very early alterations of structural (amount and structure of elastin and collagen fibres and other extracellular matrix components), as well as functional properties of arteries (modulation of vascular tone by contraction/relaxation of medial smooth muscle cells), and is considered an independent marker of cardiovascular risk [87]. Ageing and several pathological conditions (such as hypertension, smoking, and other risk factors) result in fragmentation of the elastic lamellae, distortion of their spatial organisation, increase of their stiffness due to production of glycosylation products (particularly in the case of diabetes), increased deposition of collagen, and calcification [88]. The functional component of arterial stiffness—the degree of contraction of arterial smooth muscle cells—is regulated primarily by α-adrenergic sympathetic stimulation and vasoactive substances (NO, catecholamines, angiotensin) [89]. In animal experiments, it was proven that stimulation of the α_1_-adrenoreceptors directly induces hypertrophy of the arterial smooth muscle cells and adventitial fibroblasts [90]. The increased sympathetic activity may, thus have a direct negative impact on the elastic properties of the arterial wall. Importantly, the stiffness of the arterial wall significantly affects the relationship between the blood flow and BP, cardiac afterload, BP pulsatility, the transmission of this pulsatility into the microvasculature of the target organs, and the consequent arteriosclerotic end-organ damage [91].

During recent decades, numerous methods were designed to assess arterial stiffness using several distinct techniques, evaluating various parts of the arterial tree, and differing in associations with risk factors or developed CVD [92,93,94]. For a long time, the assessment of carotid-to-femoral pulse wave velocity (PWV) has been considered the “gold standard” for the evaluation of arterial stiffness, based on a large body of epidemiological evidence highlighting the predictive ability of PWV in CVD risk estimation [87,95]. Importantly, PWV depends strongly on BP, and to a lesser extent, on the heart rate during examination [96,97]. These dependencies should be accounted for when interpreting PWV values, particularly in studies where large BP differences between subjects are present (e.g., hypertension studies) [98,99]. This issue may be particularly relevant in the case of stress-induced exaggerated cardiovascular sympathetic activity related to the clinical environment, the so-called white-coat effect, which is independently associated with female gender and inversely associated with the frequency of the clinical visits [95,98,100]. For this reason, novel and more accurate methods were developed for the evaluation of arterial stiffness that are more independent from BP.

Cardio-ankle vascular index (CAVI) is one of these methods, and reflects the overall stiffness of elastic and muscular arteries, and provides operator-independent information about the structural and functional arteriosclerotic changes [89,101]. For several years, CAVI was assumed to be fully BP-independent. However, recently, it was shown to be still slightly affected by actual BP, although to a much lesser degree than PWV [93,98,102,103]. A mathematical correction for this effect was introduced resulting in a novel index: CAVI_0_, which should, theoretically, represent a parameter of arterial stiffness that maximally suppresses the effect of short-term changes in BP [98,102]. So far, studies with CAVI_0_ support its advantage and robustness, particularly in the assessment of relatively inconspicuous arteriosclerotic alterations [98,104,105]. Among the parameters derived from PWV, CAVI and particularly CAVI_0_ have the advantage of being largely BP-independent. This removes the need for statistical blood pressure correction, whereby acute BP-dependent and -independent effects are difficult to disentangle [99]. More reference values for CAVI and CAVI_0_ for clinical applications are becoming available [105,106,107]. 

Although CAVI and CAVI_0_ have potential advantages in terms of BP resistance, carotid-femoral PWV remains an important risk marker, particularly because it exclusively evaluates the (elastic) aortic arterial bed and does not include the (more muscular) arm and leg arterial beds (that CAVI and CAVI_0_ do include), and because the proximal elastic arteries may be affected by arteriosclerosis prior to the muscular arteries [77]. Moreover, carotid-femoral PWV has already shown specific associations with cardiovascular risk factors and parameters of subclinical organ damage and is considered as an intermediate end-point for CVD due to its consistent independent predictive value for both fatal and non-fatal cardiovascular events [92,95]. 

A major rationale behind the assessment of central artery stiffness is its relationship to pulse pressure (the difference between systolic and diastolic blood pressure). Pulse pressure (in particular, central pulse pressure) is related to cardiac afterload in two ways: (1) a stiffer arterial bed with a higher pulse wave velocity results in an earlier return of the reflected pressure wave, causing blood pressure amplification; and (2) a stiffer arterial bed shows a reduced “Windkessel” effect, i.e., less capacity to buffer the pulsatile blood flow from the heart, resulting in a greater pressure fluctuation [108]. Typically, blood pressure is measured at the brachial artery using an inflatable cuff. However, brachial blood pressure systematically differs from central (aortic) BP due to pressure amplification [109]. Although central blood pressure can be directly measured invasively using a catheter, it can also be reconstructed from a peripheral (e.g., radial) blood pressure waveform using an algorithm [109,110] resulting in a non-invasive blood pressure measurement that is more representative of the blood pressure that the heart ‘sees’. In this context, Pini et al. [111] have shown a better prognostic value of central blood pressure as compared to brachial blood pressure.

However, using data from the Framingham Heart Study, it was recently shown that central pulse pressure only partially predicts cardiovascular risk, and that inclusion of PWV improves this prediction [112]. From this perspective, the current study reported a significant association between central blood pressure and PWV [113]. Nevertheless, several studies reported that after correction for confounders, only PWV (and not central pulse pressure) was associated with an increased risk for a first cardiovascular event [114,115]. The central blood pressure and PWV methodologies are summarised in a recent review [116].

To the best of our knowledge, there are no studies using central blood pressure in patients with cervical cancer or HPV infection. From this perspective, the use of central blood pressure could yield important information, especially on HPV-related cardiovascular risk.

A summary of the discussed biomarkers for early atherosclerotic changes is given in Table 1.

## 4. Conclusions

During recent years, a considerable body of evidence has accumulated demonstrating an increased risk of atherosclerotic CVD caused by HPV infection. Notwithstanding the need to further study the underlying pathophysiological mechanisms, it is the time to incorporate the current knowledge into an intensive effort to design a personalised algorithm for the prediction of cardiovascular risk in individuals with persisting HR-HPV infection, particularly in women with precancerous conditions of the cervix and cancer of the cervix. A personalised estimation of CVD risk would provide an objective evaluation of the medical conditions and minimise the risk of health complications. For the effective application of these methods in clinical practice, it is necessary to identify which biomarkers are sensitive to the effects of certain combinations of risk factors. The current diagnostic methods for the assessment of arterial stiffness and endothelial function seem to represent a promising approach in the future personalised adjustment of the commonly-used scores and schemas for the prediction of cardiovascular risk. Novel innovative diagnostic procedures aimed at decreasing the health burden associated with HPV infection and their implementation could positively affect the health of a relatively large, young population.

## Figures and Tables

**Table 1:** Biomarkers for early atherosclerotic changes.

Biomarker	Selected Major Studies	Significance
**Endothelial Function**		
Flow-mediated dilation (FMD) as an index of macrovascular endothelial function	Gori et al., 2012 (451 subjects) adults [117]	Predicting the presence of coronary artery disease in patients before coronary angiography
Peripheral arterial tonometry (PAT) as an index of reactive hyperaemia	Framingham study (Hamburg et al., 2008 (1957 subjects)) adults [118]	PAT is related to metabolic cardiovascular risk factors (smoking, cholesterol, body mass index)
**Arterial Stiffness**		
Pulse wave velocity (PWV) the current “gold standard” arterial stiffness metric	Aristizábal-Ocampo et al., 2018 (3160 subjects) adults [119]	Increased PWV values related to age and blood pressure
	Park et al., 2013 (1779 subjects) adult women [120]	Increased PWV values in women with atypical cervical cells
Central blood pressure (CBP)	Zuo et al. 2018 (675 subjects) adults [121]	CBP improved prediction of CVD compared to peripheral pressure hypertensive patients
Cardio-ankle vascular index (CAVI) $\mathrm{CAVI}=a\;.\;\underset{\mathrm{unscaled}\;\mathrm{CAVI}}{\underbrace{\left(\ln\left(\frac{P_{s}}{P_{d}}\right)\cdot \frac{{\mathrm{PWV}}^{2}\cdot 2\rho }{P_{s}-P_{d}}\right)}}+b$ [122] Cardio-ankle vascular stiffness index (CAVI_0_) ${\mathrm{CAVI}}_{0}=\frac{{\mathrm{PWV}}^{2}\cdot 2\rho }{P_{d}}-\ln\left(\frac{P_{d}}{P_{\mathrm{ref}}}\right)$ [99] ${\mathrm{CAVI}}_{0}=\frac{\mathrm{CAVI}-b}{a}\cdot \frac{\frac{P_{s}}{P_{d}}-1}{\ln\left(\frac{P_{s}}{P_{d}}\right)}-\ln\left(\frac{P_{d}}{P_{\mathrm{ref}}}\right)$ [122]	Wohlfart et al., 2017 (2160 subjects) adults [106]	Increased CAVI in smokers and patients with dyslipidaemia
	Namekata et al., 2011 (32,627 subjects) adults [107]	Group with increased cardiovascular disease (hypertension, hyperglycaemia, abnormal lipid metabolism, ischemic and sclerotic changes) showed increased CAVI
	Philip et al., 2015 (292 subjects) children [123]	Lower CAVI in overweight children suggesting vascular adaptation to obesity

*P*_s_, systolic blood pressure; *P*_d_, diastolic blood pressure; *a,b*, CAVI scale constants; PWV, pulse wave velocity, ρ = 1050 $\frac{\mathrm{kg}}{m^{3}}$ blood mass density; P_ref_, reference pressure.

## Abbreviations

HPV	human papillomavirus
CVD	cardiovascular diseases
HR-HPV	high-risk strains of human papillomavirus
DNA	deoxyribonucleic acid
BP	blood pressure
LDL	low-density lipoprotein
HDL	high-density lipoprotein
HIV	human immunodeficiency virus
EBV	Epstein-Barr virus
IMT	intima-media thickness
MetS	metabolic syndrome
SOX18	SRY-related HMG–box 18
miRNAs	micro RNAs
NO	nitric oxide
FMD	flow-mediated dilation in brachial artery
PAT	peripheral artery tonometry
PWV	pulse wave velocity
CAVI	cardio-ankle vascular index
CAVI_0_	cardio-ankle vascular stiffness index 0
CBP	central blood pressure
*P*_s_	systolic blood pressure
*P*_d_	diastolic blood pressure
a,b	CAVI constants
ρ	blood mass density
*P*_ref_	reference pressure
